# Computational approaches for network-based integrative multi-omics analysis

**DOI:** 10.3389/fmolb.2022.967205

**Published:** 2022-11-14

**Authors:** Francis E. Agamah, Jumamurat R. Bayjanov, Anna Niehues, Kelechi F. Njoku, Michelle Skelton, Gaston K. Mazandu, Thomas H. A. Ederveen, Nicola Mulder, Emile R. Chimusa, Peter A. C. 't Hoen

**Affiliations:** ^1^ Division of Human Genetics, Department of Pathology, Institute of Infectious Disease and Molecular Medicine, Faculty of Health Sciences, University of Cape Town, Cape Town, South Africa; ^2^ Computational Biology Division, Department of Integrative Biomedical Sciences, Institute of Infectious Disease and Molecular Medicine, CIDRI-Africa Wellcome Trust Centre, Faculty of Health Sciences, University of Cape Town, Cape Town, South Africa; ^3^ Center for Molecular and Biomolecular Informatics (CMBI), Radboud Institute for Molecular Life Sciences, Radboud University Medical Center, Nijmegen, Netherlands; ^4^ African Institute for Mathematical Sciences, Cape Town, South Africa; ^5^ Department of Applied Sciences, Faculty of Health and Life Sciences, Northumbria University, Newcastle, United Kingdom

**Keywords:** multi-omics, data integration, multi-modal network, machine learning, network diffusion/propagation, network causal inference

## Abstract

Advances in omics technologies allow for holistic studies into biological systems. These studies rely on integrative data analysis techniques to obtain a comprehensive view of the dynamics of cellular processes, and molecular mechanisms. Network-based integrative approaches have revolutionized multi-omics analysis by providing the framework to represent interactions between multiple different omics-layers in a graph, which may faithfully reflect the molecular wiring in a cell. Here we review network-based multi-omics/multi-modal integrative analytical approaches. We classify these approaches according to the type of omics data supported, the methods and/or algorithms implemented, their node and/or edge weighting components, and their ability to identify key nodes and subnetworks. We show how these approaches can be used to identify biomarkers, disease subtypes, crosstalk, causality, and molecular drivers of physiological and pathological mechanisms. We provide insight into the most appropriate methods and tools for research questions as showcased around the aetiology and treatment of COVID-19 that can be informed by multi-omics data integration. We conclude with an overview of challenges associated with multi-omics network-based analysis, such as reproducibility, heterogeneity, (biological) interpretability of the results, and we highlight some future directions for network-based integration.

## Introduction

Studies that implement large-scale molecular profiling techniques (-omics technologies) have increased our understanding of disease mechanisms and led to the discovery of new biological pathways, genetic loci underpinning disease progression, biomarkers, and targets for therapeutic development (Horgan and Kenny, 2011; Sun and Hu, 2016; Karczewski and Snyder, 2018). Until recently, these studies have mostly relied on single omics investigations. Dependencies between biological features and the relationships between different molecular layers (for example transcriptome, proteome, metabolome, microbiome, and lipidome) remain mostly elusive. The holistic understanding of the molecular and cellular bases of disease phenotypes and normal physiological processes requires integrated investigations of the contributions and associations between multiple (different but parallel) molecular layers driving the observed outcome. Most importantly, genetic information flows from the genome to traits and involves several molecular layers (Sun and Hu, 2016; Hasin et al., 2017). Thus, understanding the genetic architecture of complex phenotypes would involve integrating and investigating the interactions between different molecular layers (Buescher and Driggers, 2016; Hasin et al., 2017; Chakravorty et al., 2018; Zapalska-Sozoniuk et al., 2019).

Multi-omics datasets require appropriate computational methods for data integration and analysis. These methods/models implement statistical, network-based, and/or machine learning (ML) techniques on different omics layers to elucidate key omics features associated with diseases at various molecular levels and predict phenotypic traits and outcomes with increased accuracy (Ritchie et al., 2015; Bersanelli et al., 2016; Zeng and Lumley, 2018).

Based on the hypothesis that molecular features within a system establish functional connections or are part of modules to carry out processes, network-based methods offer a framework to conceptualize the complex interactions in a system as a collection of connected nodes (molecular features). They further suggest possible connections (e.g., genotype to phenotype relationships) and/or subnetworks (e.g., biological pathways) that are informative of an observed phenotype (Chakravorty et al., 2018). Therefore, network-based methods are particularly useful to assess complex interactions within multi-omics datasets and illustrate dependencies among multiple features. In addition, some network-based methods can incorporate prior information to guide the integrative analysis. For this reason, network-based methods have attracted considerable attention in multi-omics data integration around understanding disease mechanisms and drug discovery (Wu et al., 2018; Agamah et al., 2021). Previous reviews have mostly focused on the network-based analysis of single-omics data (Camacho et al., 2018; Yan et al., 2018; Zitnik et al., 2019) or different approaches toward multi-omics data integration (Cavill et al., 2016; Duruflé et al., 2021). Here, we review different integrative network-based approaches and some tools for multi-omics data analysis.

The outline of the review is as follows; we begin with a discussion on integrative multi-omics approaches, where we highlight the approaches for network-based analyses. We then discuss the different classes of methods for multi-modal network analysis. Next, we describe several network-based integrative multi-omics tools. This is followed by a discussion on the application of network-based tools to pertinent biological questions. This section provides guidance on the choice of the most appropriate network-based tools to answer a given biological question. As further examples, we show how some tools have been applied to COVID-19 research, which is currently one of the research areas benefiting from multi-omics integration approaches. Finally, we conclude with a discussion on some challenges associated with multi-omics analysis and the possible directions to mitigate such challenges.

## Integrative multi-omics approaches

**FIGURE 1:**
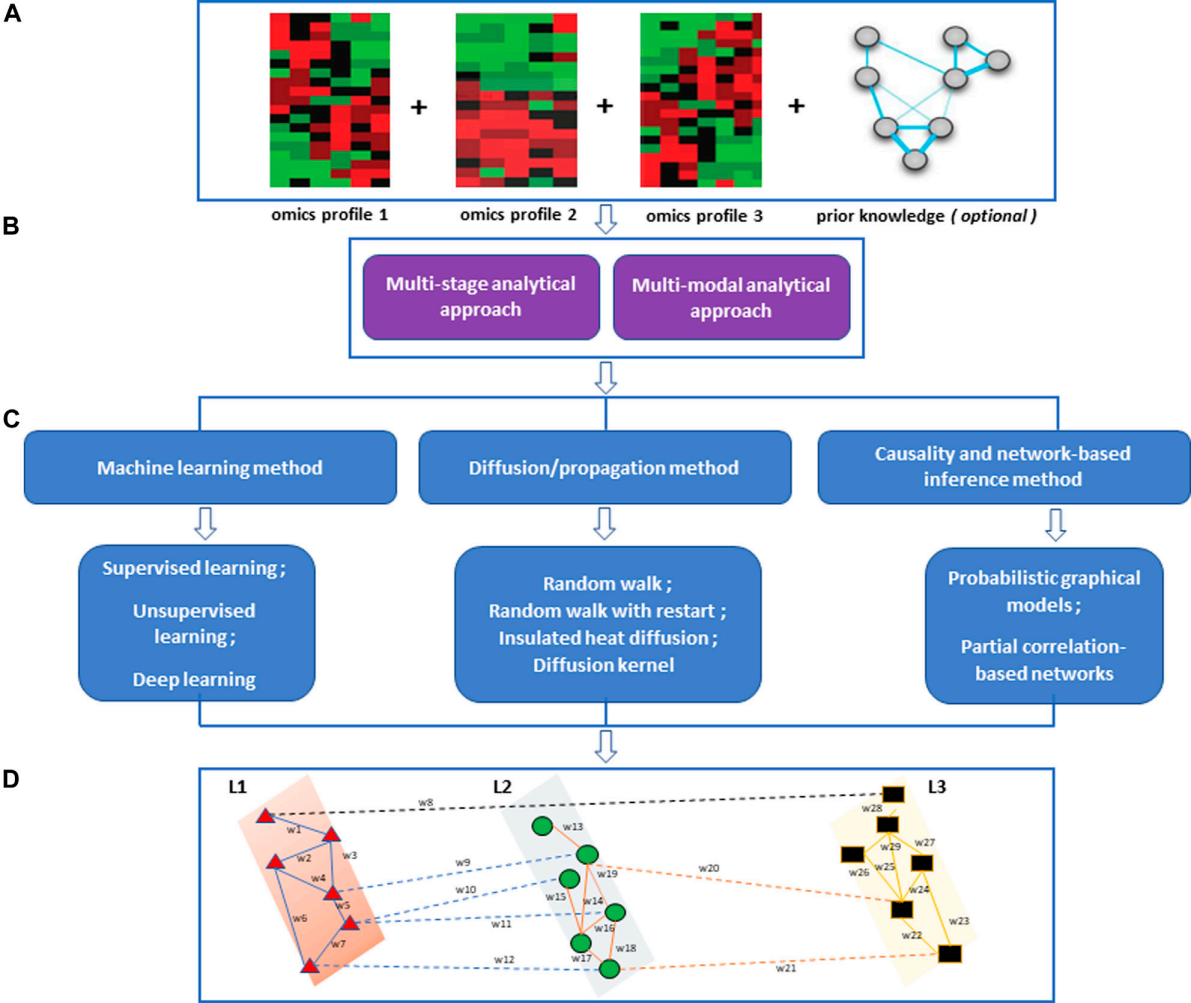
An overview of the multi-omics integration approach and the methods for network-based integration. **(A)** Processed omics data and prior knowledge for integrative analysis. **(B)** An integrative multi-omics approach that could be implemented. **(C)** Integrative network-based methods **(D)** Multi-layered network showing intra-layer interaction (solid lines) and crosstalk (dashed lines) across different layers (L1, L2, L3). The nodes are shaped and coloured to represent different omics features within the omics layers they are involved in. The edges are coloured to show different interactions within and between omics layers.

After initial data selection, processing, and quality assurance, an appropriate data analysis approach needs to be selected. We categorize integrative multi-omics analysis approaches into two main categories, multi-stage and multi-dimensional (multi-modal) analytical approaches (Figure 1) (Holzinger and Ritchie, 2012; Wen et al., 2021). The multi-stage integration involves integrating data from different technologies using a stepwise approach. In this approach, omics layers are analysed separately before investigating statistical correlations between different biological features from the datasets under consideration. This analytical approach puts an initial emphasis on the relationships of features within an omics layer and how they relate to the phenotype of interest (Ritchie et al., 2015). The multi-modal analytical approach involves integrating multiple omics profiles in a simultaneous analysis (Holzinger and Ritchie, 2012; Ritchie et al., 2015; Karczewski and Snyder, 2018; Ulfenborg, 2019).

## Methods for multi-modal network analysis

In this review, we focus on (i) machine learning-driven network-based methods, (ii) network-based diffusion/propagation methods, and (iii) causality- and network-based inference methods. The selection criteria were based on the fact that these multi-omics/multi-modal network-based methods implement network architectures together with statistical and mathematical models for integrative multi-omics data analysis. Most of these methods can be implemented in both multi-stage and multi-dimensional multi-omics analysis (Figure 1).

### Machine learning-driven network-based methods

ML is a collection of data-driven techniques for fitting an analytical model to a given dataset. ML methods do not only provide the framework to automatically learn models from large multi-omics data and make accurate predictions but also implement network architectures to exploit interaction across the different omics layers e.g., for exploring omics-phenotype associations (Reel et al., 2021). ML comprises mainly supervised and unsupervised learning methods. Supervised learning uses labelled datasets to train models to yield the desired output and emphasizes predictions by inferring discriminating rules from the data. Supervised learning model training requires comprehensive data and can be time-consuming, while unsupervised learning uses unlabelled data, to find latent structures or patterns in the data.

Classical graph-based ML methods (e.g., label propagation, a method for assigning labels to unlabelled points) can be used for a variety of tasks including generating graph edges, estimating node weights (quantitative measure of node importance) as well as estimating and optimizing edge weights (quantitative measure of the importance of the pairwise interaction between nodes) in a network to exploit the structure of graphs and learn models from the data (Karasuyama and Mamitsuka, 2017). Subsequent network optimization techniques introduce perturbations into the network and identify highly perturbed subnetworks to prioritize the most relevant features that correlate with the biological processes under study.

**FIGURE 2:**
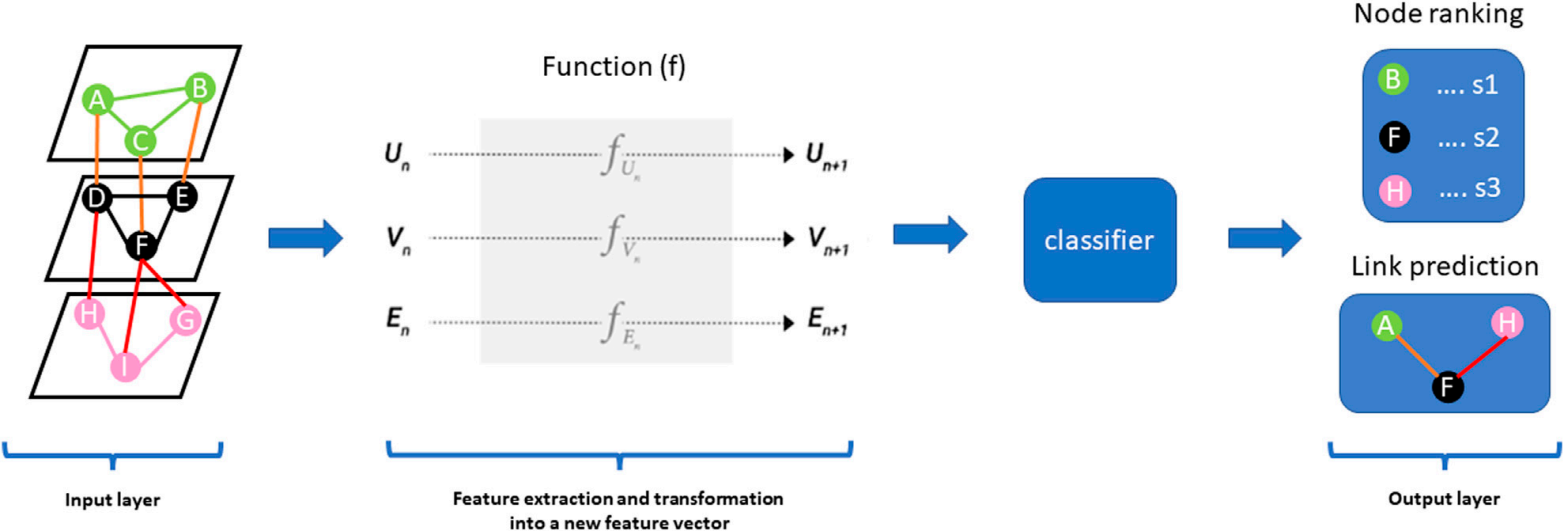
Graph Neural Networks (GNNs) are a class of deep learning methods designed to perform inference and predictions on graph data by learning embeddings for graph attributes (nodes, edges, global-context). The concept behind the architecture of these methods is such that it accepts graph data as input and produces the same input graph with updated embeddings before making predictions. GNN uses a function (f) on each graph component vector [nodes vector (Vn), edge vector (En), global-context vector (Un)] in the input graph to learn abstract feature representations of the graph to compute a new feature vector for nodes (Vn+1), edges (En+1) and global-context (Un+1)). The output layer could predict nodes ranked according to a particular score (s1, s2, s3) and also predict edges (links) in the input network.

Multiview/multi-modal ML is an emerging method for multi-omics data integration used to exploit information captured in each omics dataset and infer from the associations between the different data types (Nguyen and Wang, 2020). Multi-view learning implements the alignment-based framework and the factorization-based framework (Nguyen and Wang, 2020). The alignment-based framework is a method based on the supervised setting for seeking pairwise alignment among different omics data whereas the factorization-based framework is based on an unsupervised setting for seeking a common representation of features across different omics layers. Deep learning methods, an example of multiview/multi-modal learning, have become one of the more promising integration methods not only because of their ability to exploit the structure of graph neural networks/graph or convolutional networks in both supervised and unsupervised settings with high sensitivity, specificity, and efficiency compared to classical ML methods but also, the predictive performance and capability to capture nonlinear and hierarchical representative features (Martorell-Marugán et al., 2019; Kang et al., 2022). The hierarchical feature processing can capture complex nonlinear associations in a multi-layered manner. The architecture of deep learning models consists of the input layer, hidden layer(s), and output layer. From the perspective of multiomics data integration, most deep learning methods follow the steps of (i) feature selection, (ii) transforming high dimensional multiomics data into low-ranked latent variables, (iii) concatenating multi-omics features into a larger dataset and (iv) analysing the data for the desired task such as node ranking, link prediction, node classification and clustering (Figure 2) (Kang et al., 2022). It is worth noting that the deeper the hidden layer, the more it can learn complex patterns in the data. A major challenge for deep learning methods is the problem of overfitting due to large features and the small sample size of multi-omics data. In addition, a large amount of cleaned data is required to train and validate the model, thus influencing how the model is interpreted (Kang et al., 2022). We refer the reader to a current review on deep learning in multi-omics data integration by Kang et al. (Kang et al., 2022).

### Network-based diffusion/propagation methods

**FIGURE 3:**
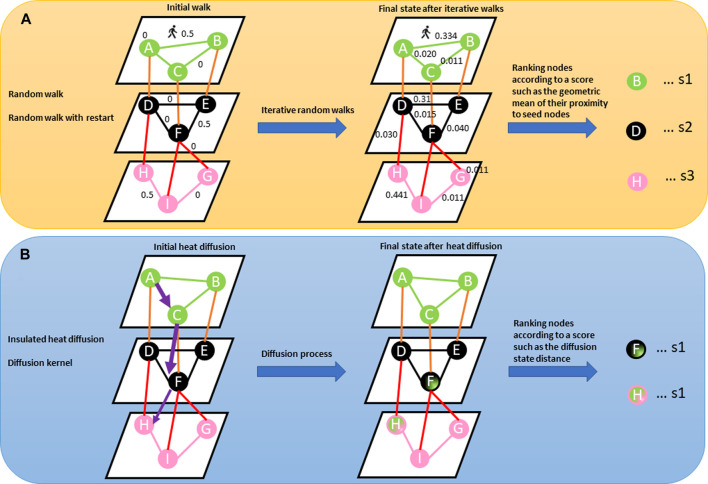
**(A)** Describes a random walk from the seed node (e.g., node A). The concept behind random walk is a guilt-by-association approach where an imaginary particle explores the network structure from seed nodes. The direction of movement of the particle is completely independent of the previous directions moved. At each step, the particle transition from any node in the graph with a certain probability (shown on the edges). The probability flow of random walks on a network is used as a proxy for information flows in the network to study the function of features, subnetworks, and prioritize features in the network. After several iterations, we are interested in the distribution of our position (Stationary distribution) in the graph (final state after iterative walks). The stationary probability distribution can be seen as a measure of the proximity between the seed(s) and all the other nodes in the graph. Nodes within the network can be prioritized using a specific metric (s1, s2, s3) such as the geometric mean of their proximity to seed nodes. **(B)** Describes heat diffusion from a reference query (e.g., node A). The concept behind heat diffusion in biological networks is perturbing nodes and simulating how the disturbance flows across edges within the network. Node disturbance means adding a scalar value (e.g., log fold changes from gene expression experiment, copy number variations) to node(s). Within a biological network, heat diffusion allows for the assessment of connectivity and topology of features which can allow the identification of relevant/dysregulated pathways and/or mutational effects across edges to neighbouring nodes. The purple arrow means diffusion jumps across different layers. The thickness of the purple arrow signifies the effect of query node (A) on nodes (F) and (H) as shown in nodes (F) and (H) in the final state graph after diffusion. Nodes within the network can be prioritized using a specific metric such as diffusion state distance.

Network-based diffusion/propagation is a technique for detecting the spread of biological information throughout the network along network edges, thanks to its ability to amplify feature associations based on the hypothesis that node proximity within a network is a measure of their relatedness and contribution to biological processes (Cowen et al., 2017; Di Nanni et al., 2020). The method has been exploited in many network-based analysis pipelines and is suitable for analysing patient-level molecular profiles with different aims including disease subtyping because of its label propagation (Di Nanni et al., 2020). Propagation methods, including random walk, random walk with restart, insulated heat diffusion, and diffusion kernel networks, provide a quantitative estimation of proximity between features associated with different data types by considering all possible paths beyond the shortest paths (Figure 3) (Cowen et al., 2017; Di Nanni et al., 2020).

From a data analysis perspective, the network diffusion (ND) methods require omics data and network data. The network data could be obtained from *a priori* knowledge, inferred from omics data, or generated using a mixed approach of *a priori* and novel knowledge (Di Nanni et al., 2020). Omics data information, e.g., genetic aberration events underlying differential expression and/or a biological phenotype, are superimposed on the nodes (source nodes) within the network before the information is propagated *via* the edges until convergence and consensus features are found (Cowen et al., 2017; Di Nanni et al., 2020).

ND methods transform input vectors of scores obtained from the omics data into dense vectors to eliminate missing values and ties. This transformation process can be applied before, after, or during the integration step to refine the results based on molecular network data (Di Nanni et al., 2020). In the ND-before integration approach, the diffusion method is applied to a collection of scores (scores obtained from the omics data) that represent the multi-omics data. The ND-after integration approach is implemented when the various multi-omics data have been initially integrated into a unique structure. The ND-during integration approach is implemented in an instance where each layer exchanges information during the diffusion process. Box 1 provides a summary of the equations related to the diffusion methods.

BOX 1 **Summary equations of the network propagation/diffusion methods**

Random Walk

$$x_{T}={\left[AD^{-1}\right]}^{k}.x_{o}$$

Random Walk with Restart (RWR)

$$x_{T}=\alpha {\left[I-\left(1-\alpha \right)\;AD^{-1}\right]}^{-1}\;.\;x_{0}$$

Insulated Heat Diffusion

$$x_{T}=\alpha {\left[I-\left(1-\alpha \right)\;AD^{-\frac{1}{2}}\right]}^{-1}\;.\;x_{0}$$

Diffusion Kernel

$${x_{T=}e}^{\alpha \left(D-A\right)}.x_{o}$$

Where,

$x_{T}$ is the final state of the network after the propagation of information throughout the network

$x_{0}$ is the initial biological information (initial state vector of aberration scores e.g., gene expression scores). A is the adjacency matrix of the network. D is the diagonal matrix of the out-degrees of nodes. $AD^{-1}$ is the normalized adjacency matrix. k is the number of time steps, α is the restart probability and I is an identity matrix

### Causality- and network-based inference methods

The mechanism of action within a biological system is fundamental to understanding such a system. For this reason, biological network inference and causal learning can be used to investigate the direct and indirect multi-layer associations and possible causal relations between omics data features in the system (Griffin et al., 2018).

Causal networks are generally graphical representations that demonstrate likely causal relations between nodes by capturing directional interactions and modelling dependencies between biological variables. The method enables researchers to put directionality between features in a network as well as decipher modules (subnetworks) and/or features associated with patient survival, disease processes, or pinpoint sources of perturbations within multi-omics biological network data (Hawe et al., 2019).

Partial correlation-based networks enable the inference of features regulating co-expression or the activities of other features within the network by estimating conditional dependencies (partial correlations) (Hawe et al., 2019). Partial correlation corrects for spurious associations among features that are mediated by other variables measured in the dataset, thereby reducing the density of the network and enhancing its interpretability (Hawe et al., 2019). These methods have been implemented to infer mechanistic regulatory interactions or predict markers in biological networks (Hawe et al., 2019).

Alternatively, network-based computational frameworks that implement probabilistic graphical models offer attractive solutions for causal reasoning and inference over multi-omics data (Friedman, 2004; Koller and Friedman, 2009; Griffin et al., 2018). A probabilistic graphical model (PGM) is a graph technique for modelling joint probability distributions and (in)dependencies over a set of random variables (Koller and Friedman, 2009). From a data analysis perspective, PGM uses graph-based representation (nodes as features and edges as direct probabilistic interactions between node pairs) as the basis to encode the complex distribution of the data for probabilistic reasoning and inference (Koller and Friedman, 2009). The framework of probabilistic graphical models includes a variety of directed and undirected models (Koller and Friedman, 2009). Directed models (e.g., Bayesian networks) require pre-defined directionality or capture conditional (in)dependencies to assert an influence on features. Undirected models (e.g., Markov networks) are undirected graphical models that offer a simpler perspective on directed models, especially in instances where the directionality of the interactions between features cannot be determined. Compared to directed models which can be used for causal reasoning and inference, undirected models are limited to inference tasks because they fail to capture the influence of nodes on neighbouring nodes.

In addition to partial correlation and probabilistic graphical models, advanced ML models and frameworks that are more computationally efficient have been explored for inferring causal relationships between multi-modal data (Peters et al., 2017; Badsha and Fu, 2019; Luo et al., 2020; Wein et al., 2021). Also, new methods that extend Bayesian networks have been developed for causal inference. For instance, Zheng et al. (Zheng et al., 2018) developed a new method to estimate the structure and inference from a Bayesian network by transforming the structure learning problem into a continuous optimization formulation that does not impose any structural assumptions on the graph. In another instance, Lachapelle et al. (Lachapelle et al., 2019) proposed a novel score-based approach to learning from Bayesian networks *via* the edge weights of neural networks. The approach developed by the authors adapts the optimization method presented by Zheng et al. (Zheng et al., 2018) to allow for non-linear relationships between variables using neural networks. Box 2 provides a summary of the equations related to the Bayesian and Markov methods. Given that the underlying principles behind network-based approaches for analysis vary, combining such approaches is feasible and may increase prediction accuracy as shown by Zheng et al. (Zheng et al., 2018) and Lacapelle et al. (Lachapelle et al., 2019).

BOX 2 **Summary equations of the Bayesian and Markov network.**

Bayesian Network

Each node in a Bayesian network is represented as a probability distribution of ***cause*** given the observed ***evidence*** which is built from the **Bayes theorem** shown below (Kotiang and Eslami, 2020).

$$P\left[Cause\;|Evidence\right]=P\left[Evidence\;|Cause\right]\;.\;\frac{P\left[Cause\right]}{P\left[Evidence\right]}$$

Thus, the full probability model for a Bayesian network is obtained by specifying the joint probability distribution (i.e., a series of the conditional probability distribution of the nodes in the network) (Kotiang and Eslami, 2020).

Markov Network

$$P\left(X=x\right)=\frac{1}{z}\exp\left(\sum_{i}\omega_{i}f_{i}\left(x_{\left\{i\right\}}\right)\right)$$

Where x is the feature vector, Z is the normalization constant calculated as

$$Z=\sum_{x\epsilon X}\exp\left(\sum_{i}\omega_{i}f_{i}\left(x_{\left\{i\right\}}\right)\right)$$

$f_{i}$ is the feature function defined as

$$f_{i}\left(x_{\left\{i\right\}}\right)=\left\{ \begin{array}{cc} 1 & F_{i}\left(x\left\{i\right\}\right)=true\\ 0 & otherwise \end{array}\right.$$

$\omega_{i}$ is the non-negative real-valued weight which reflects constraints on nodes

$F_{i}$ is the logistic formula.

## Review of network-based integrative multi-omics tools

We systematically reviewed literature primarily published between 2010 and 2022 that report on ML-driven network-based tools, network-based diffusion/propagation tools, and causality- and network-based inference tools. We further highlight the tool’s uniqueness in terms of (i) input data types, (ii) method/algorithm implemented, (iii) most important analytical steps, (iv) potential node and/or edge weighting, and (v) predicted outcome (crosstalk, disease subtypes, biomarkers, subnetworks, and patient survival). The tools presented in this review (Table 1) (i) have broad biomedical data applications and are not restricted to specific (disease) research topics only, (ii) are implemented as standalone software like R, MATLAB, Python libraries, or as part of a pipeline and, (iii) account for the weight of nodes and/or edges within the network.

**TABLE 1:** Network-based multi-omics integrative tools for predicting biomarkers, crosstalk, disease subtypes, and subnetworks/enriched modules.

Tool	Description	Major steps of the tool	Edge weighting component	Node weighting component	Outcome	Method/Approach	Input data type	year	References
**Machine learning-driven network-based tools**									
mixOmics	An R toolkit dedicated to the exploration and integration of biological data sets with a specific focus on variable selection. The package contains suite of algorithms and functions. The function network is used for graph visualization	1) Receives as input multiple matrices each representing a different omics	Infer interactions between nodes by using a pairwise association score	Leverages on measurements of variables	Relevance networks	Supervised and unsupervised ML	most omics types (genes, mRNA, metabolites, miRNomics data, proteomics)	2012	González et al. (2012)
		2) Perform network analysis using the network function							
Similarity network fusion	A network-based framework that uses networks of samples as a basis for integration. It fuses individual networks from each omics layer to represent the full spectrum of underlying data	1) SNF first creates a sample-similarity network for each omics level and then fuses these into one network using a nonlinear combination method	Uses a scaled exponential similarity kernel to determine the edge weight. The weighted edges represent pairwise sample similarities	Nodes represent samples and the node size represents a phenotype like survival	Identifies disease subtypes, performs survival prediction	Unsupervised ML	most omics types (mRNA, DNA methylation, and microRNA (miRNA) expression data)	2014	Wang et al. (2014b)
Lemon-Tree	A multi-omics module network inference software suite that finds co-expressed gene clusters and reconstructs regulatory programs involving other upstream omics data	1) Infer co-expressed gene clusters	Computes edge weight which represents the frequency with which pairs of genes belong to the same cluster	Compute the regulator score and considers the number of trees a regulator is assigned to, with what score (posterior probability), and at which level of the tree	Predicts driver genes/biomarker	Unsupervised ML	expression data, copy number, microRNA, epigenetic profiles	2015	Bonnet et al. (2015)
		2) Build consensus modules using the spectral edge clustering algorithm							
		3) Build module network							
		4) Module learning							
Multiscale Embedded Gene Co-expression Network Analysis (MEGENA)	An R package co-expression network analysis framework that effectively and efficiently constructs and analyses co-expression networks	1) Constructs fast planar filtered network	Computes a similarity score between node pair	Compute node degree as node weight/size	Predicts subnetworks, driver hubs	Unsupervised ML	Genes, mRNA, Fast planar filtered network	2015	Song and Zhang, (2015)
		2) Identify multi-scale clustering structures							
		3) Perform multiscale hub analysis							
		4) Perform cluster-trait association analysis							
Omics Integrator	The approach applies advanced network optimization algorithms to a network to find high-confidence, interpretable subnetworks that best explain the data	1) Garnet identifies a set of transcriptional factors associated with mRNA expression changes by incorporating epigenetic changes nearby expressed genes	Uses least-squares regression to relate the transcription factor affinity scores to mRNA expression changes	Transcription factors with motifs exhibiting statistically significant regression coefficients are given a weight of–log (*p*-value)	Predicts subnetworks that connect changes observed in omics data	Supervised ML	most omics types (mRNA, epigenetic changes, proteins, metabolites)	2016	Tuncbag et al. (2016)
	The software is comprised of the Garnet and Forest tools	2) Garnet scans regions proximal to transcribed genes for transcription factor binding sites and then regresses transcription factor affinity scores against gene expression changes	Forest converts uniform edge weights to costs using a scoring function	The prize function assigns negative weights to nodes based on the number of connections they have in the interactome					
	Forest provides perturbation strategies for perturbation analyses to determine the robustness of a network	3) Forest identifies a condition-specific functional sub-network from user data and a confidence-weighted interactome							
		4) The confidence-weighted interactome is integrated with the ‘omic’ hits using the prize-collecting Steiner forest algorithm, where the data is either connected directly or *via* intermediate nodes, called ‘Steiner nodes’							
Weighted Similarity Network Fusion	A method that implements a modified similarity network approach to identify disease subtypes. It accounts for feature weights when clustering patients	1) Build a regulatory network from the input data	Considers the similarity of two patients by considering the overall difference between the expression levels of all their features and the weight of each feature	Computes feature weights by first ranking features using a modified PageRank algorithm followed by Integrating feature ranking and feature variation	Identifies disease subtypes, performs survival prediction	Unsupervised ML	miRNA, mRNA, transcription factors	2016	Xu et al. (2016)
		2) Calculating the weight for each feature and ranking the features based on network information and the expression variation of the features							
		3) Obtain weighted sample similarity networks from genes (mRNAs, TFs) and miRNAs separately using the weights and expression data of the features							
		4) Perform network fusion and clustering to find patient groups that imply disease subtypes							
iOmicsPASS	A method for integrating multi-omics profile over genome-scale biological networks and identifying predictive subnetworks that provides the mechanistic interpretation of a specific phenotype	1) Integrates quantitative multi-omics data by computing interaction scores for a network	Computes scores for each molecular interaction. The scores are derived in the context of the type of interactions data (TF regulatory network and protein-protein interaction network with or without DNA copy number)	Utilizes measurement of each molecule in their respective omics data sets as node score	Predicts phenotypic group-specific subnetworks, feature selection	Supervised ML	Biological network, mRNA, proteomics data, DNA copy number, sample metainformation	2019	Koh et al. (2019)
	The tool considers molecular interactions within and between omics data types as a data feature	2) Discover molecular interactions whose joint expression patterns predict phenotypic subnetworks/groups							
		3) Report biological pathways enriched in the subnetworks using a modified nearest shrunken centroid algorithm							
Sparse CRossmodal Superlayered Neural Network (SCR-SNN)	A subtype classification model that represents a sparse version of a cross-modal super-layered neural network	1) Biomarker filtering	Estimates connection between nodes	Compute weight for nodes	Predicts disease subtype	Neural network	DNA methylation, mRNA	2020	Joshi et al. (2020)
		2) Biomarker selection, using a cross-modal, super-layered neural network							
		3) Integration of selected biomarkers from omics data							
		4) Prediction model building							
Integrative Network Fusion	A framework for high-throughput omics data integration that leverages machine learning models to extract multi-omics predictive biomarkers	1) A set of top-ranked features is extracted by juxtaposition by Random Forest (RF) and linear Support Vector Machine (LSVM) classifiers	Uses a scaled exponential Euclidean distance kernel to compute edges weight	Implements a feature ranking scheme on similarity network fusion integrated features	Identifies disease subtypes and predictive biomarkers	Supervised ML	mRNA, microRNA expression, protein levels, copy number variants, DNA Methylation	2020	Tuncbag et al., (2016); Chierici et al., (2020)
		2) A feature ranking scheme is computed on similarity network fusion-integrated features							
		3) A random forest model is trained on the intersection of two sets of top-ranked features from the juxtaposition and feature ranking scheme (rSNF) and provides compact predictive biomarkers							
Discovery of active Modules In Networks using Omics (DOMINO)	A network‐based active module identification algorithm used for identifying subnetworks that show significant over‐representation of accrued activity signal (“active modules”)	1) Receives as input a set of genes flagged as the active genes in a dataset and a network of gene interactions	Uses the confidence scores of the tissue-specific functional interactions as weights of edges	Uses gene activity scores	Predicts subnetworks	Unsupervised ML	gene network and transcriptomics data	2021	Levi et al. (2021)
		2) Partition the network into disjoint, highly connected subnetworks							
		3) Detect relevant subnetworks containing active over‐represented genes							
		4) Further, refine subnetworks into compartments							
		5) Repartition’s subnetwork compartments in putative modules							
		6) Reports final modules that are over-represented by active genes							
multi-source information super network	A network-based framework for constructing a single network from multi-source data	1) Constructs a super network based on the weighted sum of the pairwise weighted edge vectors (for each pair of genes)	Computes edge weights	Computes gene-specific scores based on characteristics and topology of the super network	Predicts subnetworks	Unsupervised ML	Genes, pathway information, CNVs, Drug data, mRNA, miRNA, PPI	2018	Zachariou et al. (2018)
i-Modern	A deep learning network framework for integrating multi-omics data	1) Feature extraction using optimized autoencoder	Estimate connection between nodes	Implements a randomization approach to explore node weight	predict omics signatures, patient subgroup classification	Neural network	miRNA, somatic mutations, copy number variation (CNV), DNA methylation, proteins	2022	Pan et al. (2022)
		2) Low-dimensional feature extraction *via* Cox-PH models							
		3) Patient subgroup classification							
OmicsNet 2.0	A network-based multi-omics analysis platform and an R package (OmicsNetR) to easily build, visualize, and analyze multi-omics networks	1) Accepts different data types as input	The methodology does not take edge directionality orweights into account	Uses feature activity scores	Predicts sub-networks, crosstalk	Unsupervised ML	Genes, proteins, transcription factors, miRNAs, metabolites, SNPs, Taxa, lc-ms Peaks	2022	Zhou et al. (2022)
		2) Search different molecular interaction database							
		3) Creates multi-omics networks							
		4) Performs network visual analytics							
multi-omics data integration for clustering to identify cancer subtypes (MDICC)	A method for multi-omics data integration that implements affinity matrix and network fusion methods	1) Construct an affinity matrix for different omics data based on a Gaussian kernel function	Computes edge weight as a measure of the Euclidean distance between samples	Utilizes measurement of each molecule in their respective omics data	Predicts disease subtypes	Unsupervised ML	mRNA, miRNA, proteomics data, DNA methylation	2022	Yang et al. (2022)
		2) Fuse affinity matrices into a new relational matrix with low rank							
		3) Cluster fused network							
**Network-based diffusion/propagation tools**									
Tied Diffusion of Interacting Events (TieDIE)	TieDIE method extends the heat diffusion strategies by leveraging different types of genomic inputs to find relevant genes on a background network with high specificity	1) Computes scores for each node in the graph	The diffusion approach is used to describe the edge score between node pairs (1 and -1). Aij = 1 if node i activates node j, Aij = −1 if node i represses or inactivates node j, and 0 otherwise, where A is an adjacency matrix	Scores between -1 and +1 are assigned to the nodes reflecting a positive or negative association with the disease state	Predicts biomarkers and disease-specific subnetworks	Diffusion-based	genes, proteins, biological pathway features, mRNA, DNA methylation	2013	Paull et al. (2013)
		2) Utilizes multiple diffusion processes to predict disease-related genes, subnetworks, and pathways		A node score of 0 reflects genes not known to be associated with the disease process					
				Nodes scores could represent experimental measurements					
Network-based Integration of Multi-omics Data (NetICS)	A gene prioritization method that is a framework for per-sample network-based integration of diverse data types on a directed functional interaction network	1) Constructs a directed functional interaction network from input functional interactions	Compute connectivity scores between node pairs	Compute a ranking score for all genes	Predicts biomarkers	Random walk	miRNA-gene interaction, mRNA, DNA methylation, genetic aberrations, protein levels	2018	Dimitrakopoulos et al. (2018)
	NetICS provides insight into how aberration events that are different between samples of the same disease type cause similar expression changes in other genes	2) Diffuse aberration scores from the aberrant genes following the directionality of the network interactions							
		3) Diffuse differential expression scores from differentially expressed genes							
		4) Predicts how aberration events cause expression changes through gene interaction							
Hierarchical HotNet	An algorithm that simultaneously combines network interactions and vertex scores to construct, identify, and rank statistically significant high-weight altered subnetworks across different omics datasets. It addresses the limitations of HotNet (Vandin et al., 2012), HotNet2 (Leiserson et al., 2015) by combating ascertainment bias in data and integrating both network topology and vertex score	1) Combines network topology and vertex scores	Defines a similarity measure between node pairs using both network topology and vertex scores	Uses vertex scores in the input network	Predicts a hierarchy of mutated subnetworks	Random walk	Interaction network with vertex scores	2018	Paull et al., (2013); Reyna et al., (2018)
		2) Defines a similarity matrix from the network using a random walk-based approach							
		3) Implements hierarchical clustering to construct a hierarchy of clusters consisting of highly connected components							
		4) Assesses the statistical significance of clusters							
regNet	regNet R package utilizes gene expression and copy number data to learn regulatory networks to estimate the potential impacts of individual gene expression alterations on clinically relevant signature genes	1) RegNet learns a regulatory network from a large collection of paired gene expression and copy number profiles	Compute a connectivity table that represents learned links between genes	Compute impact score for regulator genes, describing the contribution to expression changes in another gene	Predicts driver genes or disease biomarkers	Diffusion-based	transcription factors, mRNA, copy number data	2018	Seifert and Beyer, (2018); Marín-Llaó et al., (2020)
		2) Uses network propagation to quantify the impacts of altered genes sample-specific gene expression changes on other clinically relevant target genes							
Integrative multi-cohort and multi-omics meta-analysis framework	A multi-omics meta-analysis framework that can identify robust molecular subnetworks and biomarkers for a given disease condition	1) Module (A) takes multiple independent mRNA datasets and performs a leave-one-out meta-analysis to identify reliable differentially expressed genes	The confidence score for each protein-protein interaction is obtained from the STRING database	Utilizes experimental values from differential expression and methylation for omics features	Predicts biomarkers and subnetworks describing patients' clinical outcome	Diffusion-based	mRNA, DNA methylation, protein-protein interactions	2019	Shafi et al. (2019)
		2) Module (B) takes multiple independent DNA methylation datasets and identifies differentially methylated genes							
		3) Module (C) identifies methylation-driven genes							
		4) Methylation-driven genes are used as inputs in a network propagation algorithm to identify the proposed subnetworks							
Random walk with restart on multiplex and heterogeneous biological networks	A random walk algorithm able to exploit multiple biological interaction sources to integrate multiplex-heterogeneous networks	1) Define adjacency matrix for input networks	Generates weighted or unweighted adjacency matrix	Scores nodes according to their proximity to the seed nodes	Predicts candidate features and subnetworks	Random walk	Multi-modal data	2019	Valdeolivas et al. (2019)
		2) Compute transition probabilities of the random walk with restart							
		3) Performs propagation from seed nodes							
MultiPaths	A Python framework to build customized harmonized multi-omics networks from multiple biological databases. MultiPaths framework contains two independent Python packages: DiffuPy and DiffuPath useful for interpreting and contextualizing results from multi-omics experiments	1) DiffuPy implements four existing network propagation algorithms and five graph kernels and enables propagating user-defined labels, either as lists of entities or lists of entities with their corresponding quantitative values	The methodology does not take edge directionality or weights into account for propagation	Compute node scores using a function of graph kernel and input scores	Predicts subnetworks	Diffusion-based	genes, mRNA, metabolites, miRNomics data, biological pathway/processes data	2020	Reyna et al., (2018); Marín-Llaó et al., (2020)
		2) DiffuPath, wraps the generic diffusion algorithms from DiffuPy and applies them to construct biological networks							
Analytic and integration framework for multi-omics longitudinal datasets	An integrative framework for building multi-omics networks from longitudinal datasets. It consists of multi-omics kinetic clustering and multi-layer network-based analysis. The method is based on the modeling and clustering of expression profiles with similar behaviours using the timeOmics (Bodein et al., 2019) approach	1) Performs network reconstruction	Infers correlations between molecules based on multi-omics data	Uses experimental measurements as node scores	Identify crosstalk, key biological functions, or mechanisms	Random walk	Metabolites, genes, protein abundance, mRNA	2020	Bodein et al. (2020)
		2) Perform over-representation analysis							
Random Walk with Restart for multi-dimensional data Fusion (RWRF)	The method uses a similarity network of samples as the basis for integration	1) Construct a similarity network for each data type	Edge weight is estimated by calculating the similarity measure	Estimate stationary probability distribution which indicates similarity between the seed node and other nodes	Identify disease subtypes	Random walk with restart	mRNA, DNA methylation, microRNA	2021	Wen et al. (2021)
		2) Fuse similarity networks							
		3) Performs random walk with restart on the multiplex network							
		4) Performs network clustering							
**Causality- and network-based inference tools**									
Differential network analysis in genomics (DINGO)	DINGO is a pathway-based model for estimating patient group-specific networks and making inferences on differential network activation between patient-specific groups. DINGO jointly estimates the group-specific conditional dependencies by decomposing them into global and group-specific components	1) Estimates global component, which represents the relations common to both patient-specific groups	Constructs differential scores for group-specific edges	The vertices are ordered by their degree centrality	Predicts driver genes	Differential network approach	mRNA, DNA copy number, DNA methylation, microRNA	2015	Ha et al. (2015)
		2) Estimates local group-specific component which represents the differential unique relations in each patient-specific group							
		3) Determines significant differential edges							
Permutation-based Causal Inference Algorithms with Interventions	The non-parametric algorithm is used to learn directed acyclic graphs comprising both observational and interventional data. An example is the greedy sparsest permutation algorithm	1) Generate an interventional distribution	Estimates edge weight	Utilizes experimental measurements of features	Allows for inference of causal graphs	Unsupervised ML	Multi-modal data (omics, clinical data)	2017	Wang et al. (2017)
		2) Search for a permutation							
		3) Learn from interventions							
iDINGO	iDINGO R package is an expansion of DINGO. The package estimates group-specific dependencies between different omics data and make inferences on the integrative differential networks, considering the biological hierarchy among the omics platforms. It integrates omics data using the chain graph model	1) Integrate ordered data platforms using the chain graph model	Constructs differential scores for group-specific edges	The vertices are ordered by degrees (number of connections)	Predicts hub omics features characterized by the number of differential edges	Differential network approach	mRNA, DNA copy number, DNA methylation, microRNA	2018	Class et al. (2018)
		2) Constructs differential scores for group-specific edges to determine the significant differential edges							
prior incorporation Mixed Graphical Model (piMGM)	Can learn with accuracy the structure of probabilistic graphs over mixed data by appropriately incorporating priors from multiple sources	1) Incorporates prior information from multiple sources	Leverage conditional dependencies to estimate the strength of edges	Utilizes experimental measurements of features	Identify disease subtypes, active pathways in healthy and disease samples	Probabilistic graphical model	Multi-modal data (omics, clinical data)	2018	Ha et al., (2015); Manatakis et al. (2018)
	Identifies gene pathways associated with disease subtype	2) Score the reliability of prior information by using a weighted scheme							
		3) Merge prior information into a single prior distribution for each edge							
		4) Learning the structure of probabilistic graphs							
		5) Uses separate regularization parameters for edges with and without priors							
		6) Determine active pathways
CausalMGM	A method for learning a causal graph over variables of mixed type linked to disease diagnosis and progression	1) Learn the undirected graph over mixed data types	Leverage conditional dependencies to estimate the strength of edges	Leverages on measurements of variables	Identify causal pathways, biomarkers, and patient stratification	Probabilistic graphical model	Multi-modal data (omics, clinical data)	2019	Sedgewick et al. (2019)
		2) Perform local directionality determinations with conditional independence tests							
Multi-Omic inTegrative Analysis (MOTA)	A network-based method that uses data acquired at multiple layers from the same set of samples to rank candidate disease biomarkers	1) Builds a differential network	The weight of edges represents the partial correlation (above threshold) between node pairs	Computes an activity score (MOTA Score) for each node based on its *p*-value and its connected nodes	Predicts driver genes or disease biomarkers	Differential network approach	mRNA, metabolite, glycomics data, proteins	2020	Class et al., (2018); Fan et al., (2020)
		2) Computes partial correlation between node pairs using graphical LASSO							
		3) Calculates the differential partial correlation to determine intra-omics connections for the network							
Integrative multi-omics network-based approach (IMNA)	An integrative multi-omics framework for regulatory network analysis	1) SNP-gene mapping pairs collection	Uses the confidence scores of the tissue-specific functional interactions as edge weight	Computes signature scores for each node from different networks. Signature scores for a gene from different networks are combined and normalized to get a composite score for each gene	Identifies tissue-specific gene interaction networks and key nodes	Bayesian network approach	GWAS signals, eQTLs, epigenomic regulatory annotations, mRNA, protein interactome, and chromatin long-range interactions	2020	Chen et al. (2020)
		2) Construct SNP-gene bipartite network							
		3) Construct a functional interaction network							
		4) Computes signature score for nodes in the network							
		5) Computes composite score to provide quantitative evidence of node to evaluate the importance of the regulatory function							
		6) Perform key driver analysis on tissue-specific gene interaction networks							
MRPC	An R package that learns causal graphs and allows for inference	1) Learning the graph skeleton	Incorporates the principle of Mendelian randomization as constraints on edge direction	Utilizes experimental measurements of features	Allows for inference of causal graphs	Unsupervised ML	Genomic data, mRNA	2021	Badsha et al. (2021)
		2) Orienting edges in the skeleton							
		3) Simulating continuous and discrete data							
		4) Assessment of inferred graphs							
MIMOSA2	An R package and web application metabolic network-based tool for inferring relationships in microbiome‐metabolome data	1) Construct a community metabolic model by linking microbiome data features to reference databases	The method does not take edge directionality or weights into account	Utilizes CMP score for each feature	Allows inference of microbe-metabolite relationships and predicts disease-associated features	Unsupervised ML	Metabolite, microbiome data	2022	Noecker et al. (2022)
		2) Compute community metabolic potential (CMP) scores for each taxon, sample, and metabolite							
		3) Aggregate CMP scores at the community level							
		4) Evaluates the relationship between total CMP scores and metabolites by fitting a linear regression model							

## Research questions explored using integrative multi-omics network approaches

### Understanding how crosstalk between omics layers impacts a biological process or disease phenotype

A perturbed biological system is characterized by deviations in the behaviour of the molecules (omics data features) causing changes in crosstalk (Figure 1). These changes could become apparent in multiple (connected and dependent) omics levels and may represent a wide range of molecular events responsible for disease phenotype or impaired biological processes.

Network-based diffusion/propagation tools (described in Table 1) offer a framework to identify aberrant omics features (e.g., gene expression, somatic mutations, copy number variations, molecular subnetworks informative of disease subtype) and how their presence and activities within the network induce possible (downstream) changes that might underpin disease phenotype.

In a study to understand the molecular function of SARS-CoV-2 and SARS-CoV proteins and their interaction with the human host, Stukalov et al. (Stukalov et al., 2021) profiled the interactomes of both virus groups and investigated the effect of viral infection on the transcriptome, proteome, ubiquitinome, and phosphoproteome of a lung-derived human cell line. Functional analysis of the various biomolecules within a molecular network revealed crosstalk between the cellular processes during perturbations taking place upon infection at different omics layers and pathway levels. The authors (Stukalov et al., 2021) implemented the Hierarchical HotNet ND method to explore host-SARS-CoV-2 protein interactions during viral infection and its impact on omics levels and cell lines to understand how that could influence molecular pathways. Importantly, the group observed that the transforming growth factor beta (TGF-β) signalling pathway, known for its involvement in tissue fibrosis as one of the hallmarks of COVID-19 (Mo et al., 2020), was specifically dysregulated by SARS-CoV-2 ORF8. Further results revealed that autophagy, one of the mechanisms for controlling SARS-CoV-2 replication and monitoring the progression of viral infection (Sargazi et al., 2021), was specifically dysregulated by SARS-CoV-2 ORF3. These findings highlight the biological relevance of crosstalk and the insights it provides to understanding disease mechanisms.

### Identifying modules/subnetworks for disease or disease progression prediction/prognosis

Modular organizations within a network, characterized by clusters of neighbouring nodes highlight features that are functionally related or involved in similar activities within the system. In contrast to identifying (crosstalk of) features informative of disease mechanism, the focus here is on identifying different omics data features that cluster together to inform molecular transitions that describe disease severity level and/or disease subtypes.

Network-based tools that predict disease subtypes or subnetworks informative of a phenotype or a phenotypic group (described in Table 1) are useful for answering such questions and can help in e.g., estimating survival rates across different patient groups. Tools that implement ML and ND-based methods are useful to identify clusters in a network (see Table 1). It is noteworthy that the approach or steps, algorithms, and input data types implemented by such tools to predict subnetworks vary (as described in Table 1). In a recent application of a network-based method to COVID-19 research, Sun et al. (Sun et al., 2021a), employed MEGENA (Song and Zhang, 2015), an unsupervised ML method, to perform protein-metabolite-lipid multi-omics network analysis based on the differential co-expression (correlation between pair of omics features) of these omics data features. The network analysis indicated that tryptophan metabolism and melatonin, a metabolite related to tryptophan metabolism may contribute to molecular transitions in critical COVID-19 patients. Studies have shown that tryptophan and melatonin can improve the immune system and reduce inflammation in COVID-19, suggesting that function disorder may cause impairment to tryptophan metabolism and immune response (Essa et al., 2020; Shneider et al., 2020). Interestingly, activation of tryptophan metabolism has been clinically shown to be selectively enhanced in severe patients (Takeshita and Yamamoto, 2022). The authors further identified pathologically-relevant lipid modules which are being altered among mild COVID-19 patients.

Interestingly, connections between clusters/modules in the omics data may explain the crosstalk of biological features which are specific to the disease state and may serve as biomarkers for monitoring disease progression, treatment, and management (Yan et al., 2016; Overmyer et al., 2020; Su et al., 2020).

### Identifying candidate drivers of disease mechanisms

The contributory effect of features (nodes) within a system varies and depends on factors including but not limited to the level of feature expression or abundance, the level of interaction with other features, and the (background) state of the system. While some of these omics data features are passive (i.e., have little or no effect on system stability), others may have a significant effect on the observed phenotype.

In many biological disease-related problems, exploring relationships between multi-omics data extends beyond measuring marginal associations between features. Thus, identifying biologically relevant nodes that influence changes within the system could serve as candidate disease-related nodes responsible for an underlying phenotype (Dimitrakopoulos et al., 2018). Causal and network inference methods described in Table 1 can be implemented to explore likely causal features, potential causal relationships, and infer networks that differentiate severe disease from mild in a multi-modal network. Although causal methods provide insights into likely causal agents, investigating and confirming true causality extends beyond computational analysis to experimental validation in relevant models. Also, ML and diffusion-based methods can be used to explore candidate drivers. We describe in Table 1 some network-based tools that predict candidate disease-related nodes. In a recent COVID-19-related study, Tomazou et al. (Tomazou et al., 2021) implemented a network-based multi-omics data integration approach based on a multi-source information super-network scheme (described in Table 1) to prioritize COVID-19-related genes that could be useful as drug targets. The super network was constructed based on the weighted sum of the pairwise weighted edge vectors (for each pair of features) obtained from different sources. The method then prioritizes genes in the network by calculating a characteristic score known as the Multi-source Information Gain (MIG). Some of the genes identified by the authors include Serum Amyloid A (SAA1, SAA2, SAA3) which has been clinically verified as a sensitive biomarker in evaluating the severity and prognosis of COVID-19 (Li et al., 2020), C-reactive protein (CRP) clinically shown to be a marker of systemic inflammation associated with adverse outcomes in COVID-19 patients (Smilowitz et al., 2021), Serine proteinase inhibitor A3 (SERPINA3) shown to be a biomarker for COVID-19-related organ damage (coronary artery disease) and erythropoiesis impairment (Demichev et al., 2021), and vascular cell adhesion molecule (VCAM1) shown to be a vascular and inflammatory implicated in the inflammatory response to sever COVID-19 (Birnhuber et al., 2021).

### Drug discovery

Network-based methods that employ systematic integration of disease-specific omics profiles coupled with drug-related data (e.g., FDA-approved, experimental drugs, drug-target interactions) into a heterogeneous network have been shown to provide answers to biological questions related to drug development (Wang et al., 2014a; Vitali et al., 2016; Luo et al., 2017). In this type of network analysis, nodes could represent both omics data features and non-omics data features such as drugs, diseases, and drug targets. The edges represent the functional association between the data types such as pharmacological or phenotypic information.

The network-based view of drug discovery and development may involve multiple methods or tools at different steps. ND and ML methods have been widely implemented in this research area to make predictions (Luo et al., 2017; Tomazou et al., 2021). Predictions from such methods present an effective way to complement experimental methods with the aim of, (i) identifying drug targets, (ii) understanding the disease-drug relationship, (iii) investigating drug-target interactions, (iv) identifying potential drug candidates, (v) drug response prediction, (vi) drug-drug relations, and (vii) predict effective drug combinations. Of note, driver nodes or subnetworks as predicted by tools described in Table 1 might also inform on drug targets. An interesting application of network-based methods for drug discovery is the COVID-19 study by Tomazou et al. (Tomazou et al., 2021), whereby some of the predicted candidate compounds including dexamethasone, atorvastatin, beta-estradiol, cyclosporin-A, imatinib, and remdesivir have been found to generate promising results in clinical trials (https://clinicaltrials.gov/). We describe in Table 2, some useful integrative multi-modal network-based tools that are specifically for drug discovery.

**TABLE 2:** Useful network-based integrative multi-omics tools for drug discovery.

Tool/Method	Description	Major steps of tool	Outcome	Method/Approach	Input data type	Year	References
DTINet	A computational pipeline focuses on learning a low-dimensional vector representation of features, which accurately explains the topological properties of individual nodes in the heterogeneous network, and then makes prediction based on these representations *via* a vector space projection scheme	1) Integrates a variety of drug-related information sources to construct a heterogeneous network	Drug–target interactions	Unsupervised ML	drug-related information protein-protein interactome	2017	Luo et al. (2017)
		2) Applies a compact feature learning algorithm to obtain a low-dimensional vector representation of the features					
		3) Finds the best projection from drug space onto protein space					
		4) Infers new drug-target interactions					
DrugComboExplorer	A tool for identifying driver signalling pathways and inferring the polypharmacy efficacies and synergy mechanisms through drug functional module-induced regulation of target expression analysis	1) Identify the seed (driver) genes	Prioritize synergistic drug combinations, Uncover potential mechanisms of drug synergy	Unsupervised ML	DNA sequencing, gene copy number, DNA methylation, RNA-seq data	2019	Huang et al. (2019)
		2) Explore networks from the seed genes by integrating the RNA-seq profiles and pathway knowledge					
		3) Explore networks from the seed genes by integrating the methylation profiles and pathway data					
		4) Combine the networks generated from the RNA-seq data and the methylation data					
Reciprocal nearest neighbour and contextual information encoding (RNCE)	A network integration approach accounting for network structure by a reciprocal nearest neighbour and contextual information encoding (RNCE) approach	1) Applies the similarity network fusion (SNF) approach to fuse drug networks	Predicts drug targets, drug mechanism of action	Unsupervised ML	Pharmacogenomic data such as gene expression data under drug perturbation or drug sensitivity data at the cell-line level	2021	Chen and Wong, (2021)
		2) Generate contextual information network					
		3) Compensate for the contextual information network with the initial SNF network					

## Current challenges and recommendations

### Design of experiment

The choice of a network-based integration method does not only depend on the biological question but also the experimental design. Certain network-based methods can only deal with paired data, whereas others can also deal with sparse datasets where there is no or only partial overlap between the samples profiled with the different omics layers. Importantly, the scope of the research will inform the type of data that should be generated. For instance, the paired data, herein referring to different omics data measurements from the same biological sample, is preferred when establishing a holistic picture of systems biology underpinning molecular mechanisms linked to disorders, whereas non-paired data (data generated from different biological samples) is more appropriate for comparative (meta)analysis of samples or omics data measurements. It is therefore recommended to consider the scope of research and the network-based methods that fit.

### Reproducibility

Researchers routinely expect that results generated by applying network models are reproducible. For network-based methods, the key issues related to reproducibility are non-harmonized data, biased model evaluation, and lack of transferable code or software. First, multi-omics network-based integration involves the use of heterogeneous data, and some sort of data harmonization is required. A promising approach to harmonize multi-omics research is to ensure that the data comply with FAIR data principles (findability, accessibility, interoperability, and reusability). The data FAIRification process ensures that a (meta)data schema/method which captures relations between (omics) measurements, data structure, and concepts are clearly defined and easily interpretable by both humans and computers. The metadata schema provides information about the omics data structure and facilitates easy mapping of measured features onto persistent identifiers and established biological networks to investigate the connection between network elements (Krassowski et al., 2020). Second, confidence in multi-omics network-based methods requires systematic evaluation and validation of both datasets and models as a prerequisite for benchmarking toward reproducibility (Krassowski et al., 2020). This approach requires harmonized datasets of quality and quantity that provide unbiased ground truth to ensure that the model at least predicts biologically verified features or edges. Given that there is no gold standard metric for validation, it is critical to validate on a variety of data sources and use metrics that are robust to the level of missing data. Third, to replicate results from previous studies, a detailed report of the analysis together with executable analysis code is important to achieve this purpose. The report and code could be hosted in repositories (e.g., GitHub, Bitbucket, GitLab), reproducible scientific workflow management systems (e.g., Nextflow, Galaxy), environment sharing avenues (e.g., Conda, Docker), or packaged as libraries for programming languages (Canzler et al., 2020). In addition to the key issues, adapting general best practices in the computational analysis will aid reproducibility.

### Heterogeneity

Heterogeneity (a measure of variation) of multi-omics datasets, characterized by diverse data sources, data types, and data structure results in computational complexity, analysis bias, and hampers a robust and reproducible integrative network analysis (Lee et al., 2021). There is an increasing awareness of controlling heterogeneity across multi-omics integrative analysis, but most of them are focused on paired data rather than non-paired data.

In the context of network-based integrative analysis developing models and algorithms that could account for non-uniformity by identifying the most robust signals encompassing data, heterogeneity is important. This could be in the form of variable selection models to identify important covariates with the strength of multiple datasets, and yet maintain the flexibility of variable selection between the datasets to account for the data heterogeneity (Lima et al., 2020).

### (Biological) Interpretation of results

Interpreting results from an integrative multi-omics analysis is a process of disentangling multiple functional relationships. Primarily, the systematic interpretation of results depends on the kind of biological question and the type of omics measurements used for the analysis. Different omics technologies may have different levels of completeness and sensitivity in terms of detecting biological features. This might result in some omics data types containing more information than others as well as impact the results significantly (Jung et al., 2020). It is important to consider the inherent relationship between the omics profiles used during the interpretation of the results. More often functional annotation of features is based on generalized information which allows a less comprehensive understanding of the molecular mechanisms underlying a phenotype. For this reason, incorporating relevant contextualized pathway information (e.g., tissue-specific or cell-specific) in the analysis has been useful to assess the functional relevance of nodes and subnetworks on the disease/phenotypic landscape, thereby facilitating interpretation.

The capacity to interpret predicted features and interactions of known biological relevance may take the form of deductive reasoning or semantic similarities to support a hypothesis (Guo et al., 2022). In the context of algorithms, robust node weighting and edge weighting metrics measured based on known evidence (e.g., text mining, contextualized pathway information) is important to make an inference that is potentially biologically grounded and experimentally confirmable, knowing that the association between omics layers extends from one-to-one and one-to-many to many-to-many.

### Sparsity

There is sparsity at the sample level (not all samples have been profiled with the same assays) and at the feature level. The latter is far more prominent in metabolomics and proteomics than in DNA and RNA sequencing. This is mainly due to the selection of peaks (intensities observed in MS1 survey scans) for fragmentation by data-dependent acquisition (DDA) or data-independent acquisition (DIA) tandem mass spectrometry (LC-MS/MS) approaches (Guo and Huan, 2020; Davies et al., 2021). Typically, an ideal acquisition mode ought to produce spectra of high quality for as many of the ions present in the sample as possible, however, that is not the case, resulting in sparsity at the feature level. This issue is partly but not completely resolved in the newer DIA and integrated DDA-DIA modes which operate in a less-selective manner and have higher coverage as compared to the older DDA mode (Sun et al., 2021b; Davies et al., 2021).

Another contributing factor to sparsity in omics data in tandem with omics technologies is the absence of accumulation of a molecule to a detectable level by omics platforms (evidenced even across platforms of the same omics technology (*e.g.,* next-generation RNA and DNA sequencing). This is partly associated with experimental design, poor biological sample quality, and sample processing.

For computational analysis purposes, mputation can be used to solve missing value problems; however, imputation does not apply to all omics data types (Folch-Fortuny et al., 2015). In addition to imputation, sample similarity measurement methods such as matrix calibration (Li, 2015) and the Mahalanobis distance approach (Sitaram et al., 2015) could be useful to extrapolate for missing values, however, these methods are also limited to specific omics data types. Thus, a feature may have values only in a small percentage of samples leading to sparse matrices, where features may have a wide variety of distributions. Some multi-omics data integration methods can handle sparse data and also feature reduction methods; however, skewed estimates might result in a biased interpretation of results (Greenland et al., 2016). To address the issue of sparsity in the context of networks, network integration aggregates independent data sources to form a more comprehensive attributed interactome, where the edges are qualified by specific semantic relations or similarity correlation, and the level of confidence in the node pair relationship based on evidence from similarity scores, literature and graph databases (Guo et al., 2022). Also, incorporating autoencoders, a deep learning approach, and its denoising and variational variants autoencoders (e.g., sparse autoencoders) have been used to address this issue in graph neural networks (Ng, 2011). Autoencoders learn a representation of the data from the input layer, enforce sparsity constraints and try to reproduce it at the output layer. During this process, the model can learn from incomplete data and generate new plausible values for imputation (Pereira et al., 2020).

## Future directions

An area of prospect for integrative multi-omics network-based research, which remains an important opportunity, is making efforts to limit the challenges linked with network-based multi-omics integration in the context of heterogeneity, reproducibility, sparsity, and interpretation of results as discussed above. Another area of importance is building hybrid integrative models which are capable of handling paired and non-paired omics data, as well as other biomedical data. Furthermore, efforts to develop a framework tool or metadata schema that standardizes or harmonizes various multi-omics approaches for data integration could be useful. For example, such a framework may leverage an optimized approach to weigh and prioritize genes, pathways, biological processes, drug targets, and relationships between various other biological features from the multi-omics datasets. However, such framework tools will also require the expertise of domain experts, as well as the detailed and uniform characterization of statistical and technical attributes of the data (Krassowski et al., 2020).

## Discussion

Network-based integrative multi-omics analysis offers the opportunity to elucidate interactions that can occur among all classes of molecules in a biological system as well as information flow between and within multiple omics levels. In addition, it potentially provides substantial improvement of biological understanding by helping in the interpretation of results, as compared to single omics analysis, although collecting multi-omics data from different sources does not guarantee that it will be possible to learn about (all of) the relationships present.

**FIGURE 4:**
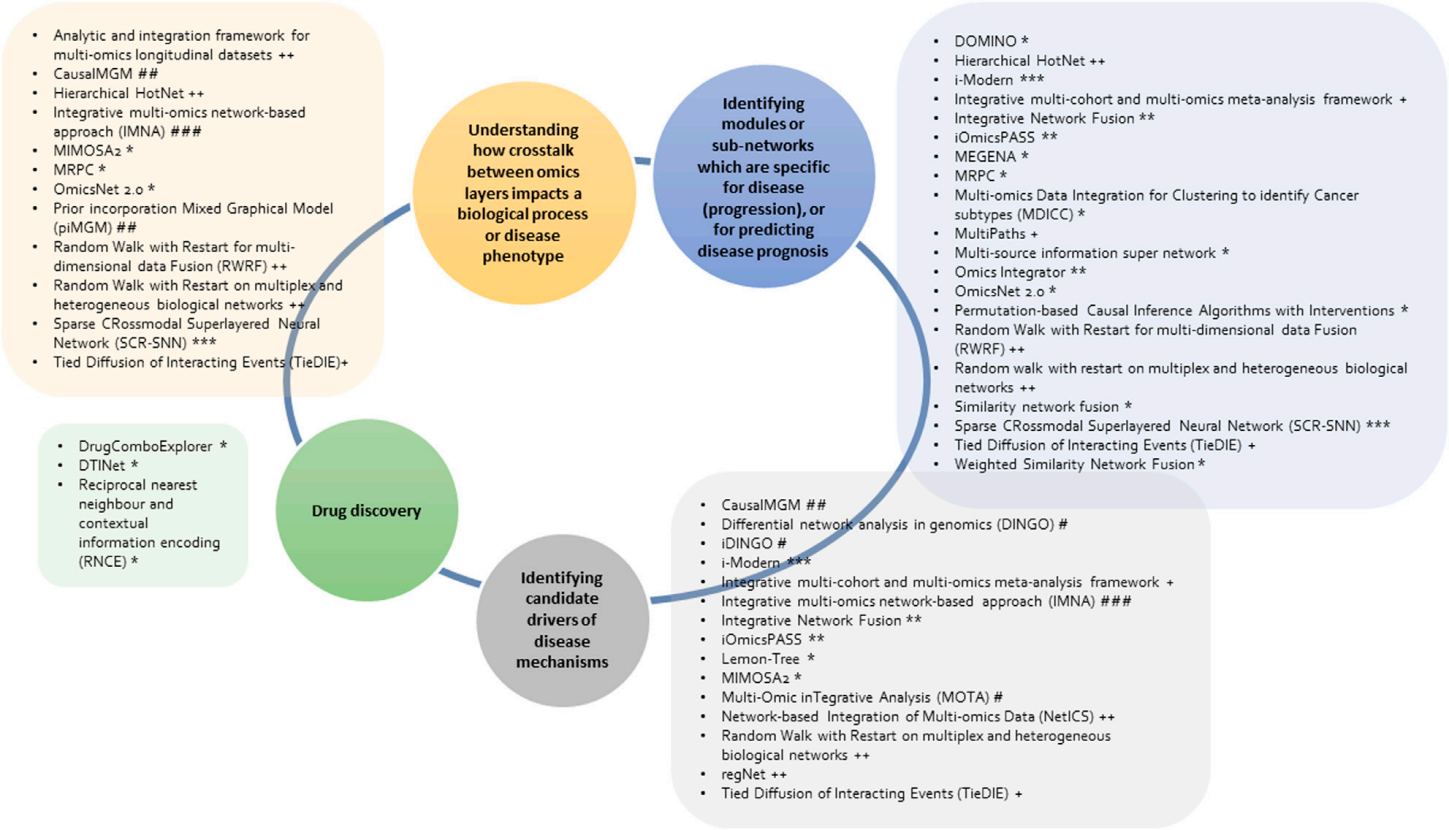
Overview of the discussed network-based multi-omics integrative tools and research questions (in the circle) that they can be applied to. The tools implement different methods including unsupervised machine learning (*), supervised machine learning (**), neural networks (***), diffusion-based (+), random walk (++), differential network (#), probabilistic graphical model (##) and Bayesian methods (###).

Various graph-based multi-omics methods have been developed for network analysis; however, their application is dependent on the scope of the research question of interest and the (omics) data types available. Consequently, this will inform the choice of an integrative analytical approach and tools. The network-based methods discussed use different scoring metrics, algorithms, and data types which together translate into a comprehensive data source/graph to be employed for interpretation into biological knowledge. The overviewand description of the tools for network-based integrative analysis (Table 1) show that different approaches can be implemented in different ways to achieve similar results. Additionally, the classification of tools (Figure 4) highlights that some tools can be applied to more than one research question. However, due to the difference in approaches of these methods, we recommend the use of multiple analytical and methodological approaches during integrative data analysis, to compare and validate the study results in different ways before interpretation for further downstream tests or follow-up studies.
